# Effect of Electro-Acupuncture on Lateralization of the Human Swallowing Motor Cortex Excitability by Navigation-Transcranial Magnetic Stimulation-Electromyography

**DOI:** 10.3389/fnbeh.2022.808789

**Published:** 2022-02-24

**Authors:** Xiaorong Tang, Mindong Xu, Jiayi Zhao, Jiahui Shi, Yingyu Zi, Jianlu Wu, Jing Xu, Yanling Yu, LuLu Yao, Jiayin Ou, Yitong Li, Shuqi Yao, Hang Lv, Liming Lu, Nenggui Xu, Lin Wang

**Affiliations:** ^1^South China Research Center for Acupuncture and Moxibustion, Medical College of Acu-Moxi and Rehabilitation, Guangzhou University of Chinese Medicine, Guangzhou, China; ^2^Nanfang Hospital, Southern Medical University, Guangzhou, China; ^3^Shenzhen Maternity and Child Healthcare Hospital, Shenzhen, China; ^4^Department of Integration of Chinese and Western Medicine, School of Basic Medical Sciences, Peking University, Beijing, China

**Keywords:** lateralization, swallowing, single-pulse TMS, resting motor threshold, motor evoked potential, electro-acupuncture (EA)

## Abstract

**Background:**

The use of transcranial magnetic stimulation combined with electromyography for the functional evaluation of the cerebral cortex in both clinical and non-clinical populations is becoming increasingly common. Numerous studies have shown that electro-acupuncture (EA) can regulate cerebral cortical excitability. However, the effect of EA on the lateralization of the human swallowing motor cortex excitability is not yet fully understood.

**Objective:**

The aim of this study was to assess whether lateralization is present in the swallowing motor cortex of healthy subjects, and to investigate the impact of EA at Lianquan (CV23) and Fengfu (GV16) on lateralization.

**Methods:**

Forty subjects were randomized 1:1 into the EA group and the sham-EA group. The bilateral swallowing motor cortices was located by a neuroimaging navigation system. Then, the resting motor threshold (RMT) and motor evoked potential (MEP) of the mylohyoid of healthy subjects were recorded while applying combined transcranial magnetic stimulation and electromyography before and after EA or sham-EA.

**Results:**

First, the RMT and MEP latency of the contralateral mylohyoid innervated by the right swallowing cortex (71.50 ± 1.67%, 8.30 ± 0.06 ms) were lower than those innervated by the left (79.38 ± 1.27%, 8.40 ± 0.06 ms). Second, EA at CV23 and GV16 reduced the bilateral RMT and enhanced the bilateral MEP latency and amplitude (*P* = 0.005, *P* < 0.001; *P* = 0.002, *P* = 0.001; *P* = 0.002, *P* = 0.009), while sham-EA did not (*P* > 0.05). Third, EA had an effect on the RMT and MEP latency in terms of lateralization changes, but this was not significant (*P* = 0.067, *P* = 0.156).

**Conclusion:**

The right swallowing motor cortex of healthy subjects is more excitable than that of the left at resting state. Thus, we found that lateralization is present in the swallowing motor cortex of healthy people, which might indicate a hemispheric dominance of swallowing predominates in the right swallowing motor cortex. In addition, EA at CV23 and GV16 can instantly promote the excitability of the bilateral swallowing motor cortices. But there was no significant difference in EA stimulation in terms of lateralization.

## Graphical Abstract

Schematic diagram of research.

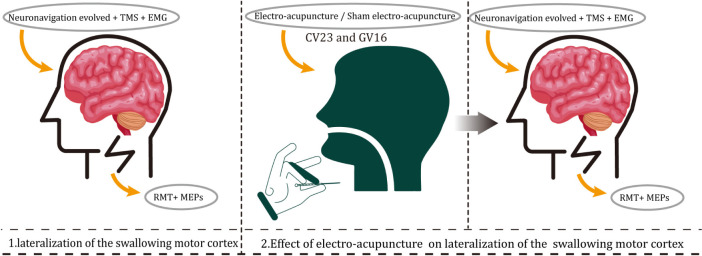

## Highlights

- The right swallowing motor cortex of healthy people is more excitable than the left.
- EA promoted swallowing motor cortex excitability, bilaterally.
- The above research results will help people understand the way of brain swallowing control more comprehensively, which may help clinicians formulate scientific rehabilitation treatment plans.

## Introduction

We have a favorite hand for writing, a preferred foot for kicking a ball, and even a favorite way to turn our head when kissing (Carey et al., 2001; Gunturkun, 2003; Papadatou-Pastou and Tomprou, 2015). These are the result of structural or functional differences between the left and right hemispheres known as hemispheric lateralization, which is a feature found in almost all major nervous systems of the human brain (Gunturkun and Ocklenburg, 2017). At the structural level, hemispheric lateralization can manifest as asymmetric gene expression in specific brain regions, asymmetry in the size and shape of brain regions, and asymmetry in the shape and structure of functional networks and the two hemispheres themselves (Crow, 2013; Caeyenberghs and Leemans, 2014; Karlebach and Francks, 2015; Ocklenburg et al., 2016). At the functional level, the most significant asymmetry in brain activity is found in language systems, as well as visual spatial processing, auditory processing neurons, memory, motor systems, and emotional processing (Goodale, 1988; Cabeza, 2002; Tervaniemi and Hugdahl, 2003; Grimshaw and Carmel, 2014). Although hemispheric lateralization is ubiquitous in the human nervous system and body, bringing about a series of highly relevant behavioral and cognitive changes, this core principle has still not been thoroughly studied.

Swallowing is one of the most complex and closely coordinated physiological activities of humans. In contrast to most somatic functions, swallowing has bilateral cerebral representation. Previous studies had found that left hemisphere damage and right hemisphere damage may be associated with different types of swallowing behaviors (Robbins et al., 1993; Daniels et al., 1996). Dysphagia would occur if damage had affected the side of the brain with the largest or dominant projection (Singh and Hamdy, 2006). This means that the functional asymmetry of the swallowing motor cortex may be important for determining the severity of the dysphagia and the recovery of the dysphagia. Hamdy et al. (1996) and Scoppa et al. (2020) identified the sites of cortical activation during swallowing using functional magnetic resonance imaging (MRI) techniques, and they found it mainly concentrated in the precentral gyrus. Therefore, similar to previous studies, our study selected the swallowing area of the precentral gyrus to measure the excitability of the swallowing motor cortex (Hamdy et al., 1997, 1998; Singh and Hamdy, 2006).

Transcranial magnetic stimulation (TMS) is an approach that allows non-invasive stimulation of neurons using time-varying magnetic fields (Halko et al., 2013). TMS has been used to detect cerebral cortex excitability (Badawy et al., 2012). The MEP elicited in peripheral muscles by TMS over human motor cortex is one of the hallmark measures for non-invasive quantification of cortical and spinal excitability in cognitive and clinical neuroscience (Bestmann and Krakauer, 2015). A series of previous studies have shown that the threshold for producing an motor evoked potentials (MEPs) reflects the excitability of a central core of neurons and MEPs indicates the excitatory state changes in the cortex (Leocani et al., 2000; Hallett, 2007; Biabani et al., 2021). As for the area of the swallowing cortex, there are many previous studies using MEPs to assess the excitability of the contralateral swallowing cortex (Hamdy et al., 1997, 1998).

Electro-acupuncture (EA) is a technique based on the traditional acupuncture method combined with modern electrotherapy. EA can stimulate nerves, and the resulting nerve impulses can strengthen the corresponding neural reflexes (Deng and Wu, 2017). Systematic review and meta-analysis have supported the claim that acupuncture therapies cannot cure dysphagia, but it can partially improve the swallowing function of patients with dysphagia, and CV23 and GV16 were the most commonly used acupoints (Lu et al., 2021; Zhong et al., 2021). However, the mechanism underlying the curative effect of EA is unknown.

As for the lateralization, Hamdy et al. (1996) illustrated that the swallowing musculature is discretely and somatotopically represented on the motor and premotor cortex of both hemispheres and interhemispheric asymmetry varied among mylohyoid, pharynx and esophagus by TMS combined with EMG. Mosier et al. (1999) showed that right hemispheric dominance showed stronger swallowing lateralization than the left hemisphere with functional MRI imaging. Besides, Rotenberg et al. (2010) demonstrated that lateralized cortical stimulation resulting in selective activation of one forelimb contralateral to the site of stimulation could be achieved by TMS using MEPs in the rat. But, Ferrari et al. (2017) failed to demonstrate lateralization in the dorsolateral prefrontal cortex by using TMS. Khamnei et al. (2019) have discovered that the dominant chewing side may originate in the dominant hemisphere of the brain (dominant hemisphere is defined by handedness) by using surface electromyography recording from masseter muscles. However, researchers failed to identify a consistent pattern of lateralization from swallowing musculature. Thus, to further explore the hemispheric dominance of swallowing function at resting state, we investigated the lateralization of the human swallowing motor cortex excitability in healthy subjects using the Resting motor threshold (RMT) and MEPs induced by TMS. More importantly, we examined the effect of EA on swallowing motor cortex excitability.

## Materials and Methods

### Study Design and Subjects

This was a single-blind randomized controlled trial. Recruitment of eligible healthy subjects was conducted at the South China Research Center for Acupuncture and Moxibustion. The protocol of this trial has been published previously (Li et al., 2019). This trial was following the Declaration of Helsinki and registered with the Chinese Clinical Trial Registry (ChiCTR-IOR-17011359). Ethical approval was obtained from the China Ethics Committee of Registering Clinical Trials (ChiECRCT-20170038). Forty healthy subjects (20 female and 20 males; age: 21.65 ± 0.28 years; body mass index: 20.40 ± 0.34; Mean Mini-mental State Examination score: 30; Kubota Water Swallow Test: Grade 1) were enrolled in the study. Subjects had no history of neurological or psychiatric disease and were not taking drugs that act on the central nervous system. Subjects gave written informed consent.

### Randomization and Blinding

Eligible subjects were randomly assigned to receive either EA or sham-EA *via* a computer randomization program (PEMS3.1, Sichuan University, Sichuan, China) using a 1:1 ratio. Due to the specific nature of acupuncture, the acupuncturist was not blinded to treatment allocation. The participants, outcome assessors, and statisticians were blinded to treatment allocation.

### Intervention

Lianquan (CV23) is an acupuncture point on the anterior part of the surface of the neck directly superior to the laryngeal prominence, in the depression at the upper margin of the hyoid bone. It is at the base of the tongue and level with the pharynx (Li et al., 2019). CV23 is also located where the mylohyoid (which is a paired muscle running from the hyoid bone to the mandible and which forms the floor of the mouth) inserts into the hyoid (Li et al., 2019; Supplementary Appendix 1). For the EA group, two sterile acupuncture needles (length: 25 mm, diameter: 0.30 mm; Huatuo, Suzhou Medical Supplies Factory Co., Ltd., Suzhou, China) were inserted into CV23 and GV16 to a depth of 0.5–1 cun. The acupuncture needles were connected to an acupuncture point nerve stimulator (HANS-200A) with a frequency of 2 Hz for 15 min, and the intensity of EA was set to the maximum-tolerated intensity of each subject (0.9–3.0 mA). For the sham-EA group, Streitberger placebo needles (Huatuo, Suzhou Medical Supplies Factory Co., Ltd., Suzhou, China) were inserted into CV23 and GV16. The Streitberger placebo needle set was invented by Streitberger and Kleinhenz (1998), and is a validated and reliable single-blind acupuncture needle used to investigate the effects of acupuncture.

### Navigation

The subjects’ brains were scanned using an MRI scanner (Signa EXCITE 3.0T HD, IGE, Milwaukee, United States) at Guangdong Provincial Hospital of Chinese Medicine. The MRI T1 file was imported into the Brainsight TMS Navigation system (Brainsight 2.3.3.dmg) and a three-dimensional brain was then reconstructed. Targets with 3 × 3 square grid were built over the swallowing motor cortex of the three-dimensional brain. The Polaris System was used to collect cortical topographical landmarks from the head of subject, which allowed the external near-infrared system to follow the figure-of-eight TMS coil in real-time. Subjects sited on the treatment couch facing the neuroimaging navigation system and TMS machine to enable location of the correct brain regions. They were asked to remain as relaxed as possible and to avoid swallowing, coughing, or vocalizing during the stimulation procedure.

### Transcranial Magnetic Stimulation and Electromyography

Transcranial magnetic stimulation was applied to the motor cortex in the bilateral hemisphere with a Magstim Super Rapid magnetic stimulator (Magstim Company, Dyfed, United Kingdom) equipped with a figure-of-eight coil (external wing diameter, 70 mm). The coil was orientated at 45°oblique to the sagittal plane. That was to ensure that the stimulus can be applied vertically to the swallowing area of the precentral gyrus. RMT and MEPs induced by TMS were recorded from the bilateral mylohyoid.

Motor evoked potentials were electromyographic signals produced by the peripheral muscles under transcranial magnetic stimulation (Peng et al., 2020). The recording electrodes were two pairs of bipolar silver-silver chloride electrodes (10 mm diameter 1.5 m cable 12/package, DIN Style, Nicolet, United States), which were placed bilaterally on each side of the mylohyoid after skin disinfection to record MEPs of the mylohyoid. The reference electrodes were placed directly next to them (1 cm). Two pairs of ground wire disk electrodes (1.25 m cable, 1/package, DIN Style) were placed 2 cm from both corners of the mouth to reduce interference to the electromyographic signal. All electrodes were checked every 15 min to ensure that they were in contact with the skin and underlying muscles.

Cortical stimulation was performed over the left and right swallowing motor cortices every 30 s according to the targets with 3 × 3 square grid, described above. First, a preliminary study was performed using initial stimulation intensities of 1.3–2.0 tesla (60–90% stimulator output) on the targets with 3*3 square grid according to tolerance degree of different subjects. This allowed the sites of square gird evoking maximal MEPs for the mylohyoid to be identified the optimal stimulation point. Next, cortical stimulation was reapplied at the optimal stimulation point using an intensity of 0.7 tesla and increased in 0.1 tesla steps until an intensity was found that MEPs of greater than 30 μV, on at least five of ten consecutive trials. The minimum stimulus intensity was defined as the RMT. Then, we used a stimulation intensity of 110% RMT to stimulate the optimal stimulation points of left and right swallowing motor cortices every 30 s during three stimulation trials, and recorded the MEPs amplitude/latency (Hamdy et al., 1996). Finally, we averaged the three MEPs recorded each time and used the averaged value as the final data for analysis (Hamdy et al., 1996).

### Statistical Analysis

Statistical analysis were performed by using the Statistical Package for Social Science (SPSS) version 20.0 (SPSS Inc., Chicago, IL, United States).χ^2^ test for categorical variables, *t*-test for continuous variables with normal distribution, and non-parametric test (Mann–Whitney *U* tests) for skewed distribution were used to detect difference in baseline characteristics between the two groups. For the outcome analysis, Mann–Whitney *U* tests was used to assess the difference between two groups, and Wilcoxon’s tests was used to assess the changes of same groups after intervention. The level of significance was set at 5% in the comparison, and all statistical testing was 2-sided. RMT/MEP lateralization was calculated according to the following formula: RMT/MEP lateralization = left swallowing motor cortex RMT/MEP – right swallowing motor cortex RMT/MEP.

## Results

Forty subjects completed the study. None of the subjects reported any persistent complaints of weakness or paresthesia after the prolonged stimulus. The study process can be found in Supplementary Appendix 2.

### Lateralization of Swallowing Motor Cortex Excitability

**FIGURE 1:**
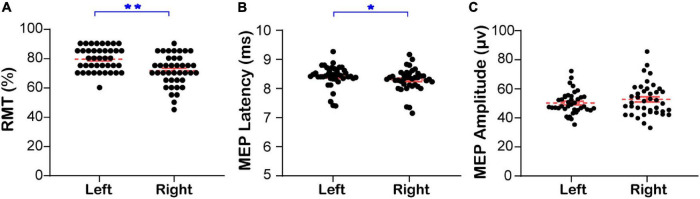
The RMT and MEP of the contralateral mylohyoid innervated by the bilateral swallowing motor cortices. **(A)** The bilateral resting motor threshold. **(B)** The bilateral latency of motor evoked potential. **(C)** The bilateral amplitude of motor evoked potential. Left: The RMT and MEP of the contralateral mylohyoid innervated by the left swallowing motor cortex; Right: The RMT and MEP of the contralateral mylohyoid innervated by the right swallowing motor cortex; RMT, resting motor threshold; MEP, motor evoked potential;*P < 0.05, **P < 0.01.

The RMT and MEPs of the contralateral mylohyoid innervated by the bilateral swallowing motor cortices of healthy subjects at resting state are shown in Figure 1 and Supplementary Appendix Table 3A, respectively. The RMT of the contralateral mylohyoid innervated by the right swallowing motor cortex (71.50 ± 1.67) was lower than that innervated by the left (79.38 ± 1.27, Mann–Whitney *U* test, *Z* = −3.859, *P* < 0.001, Figure 1A). The MEP latency of the contralateral mylohyoid innervated by the right swallowing motor cortex (8.30 ± 0.06) was shorter than that innervated by the left (8.40 ± 0.06, Mann–Whitney *U* test, *Z* = −2.041, *P* = 0.041, Figure 1B). However, there was no significant difference in the MEP amplitude (R: 52.68 ± 1.76, L: 50.15 ± 1.23, Mann–Whitney *U* test, *Z* = 0.804, *P* = 0.422, Figure 1C). Thus, the excitability of the right swallowing motor cortex was higher than that of the left swallowing motor cortex.

### Effect of Electro-Acupuncture on Swallowing Motor Cortex Excitability

**FIGURE 2:**
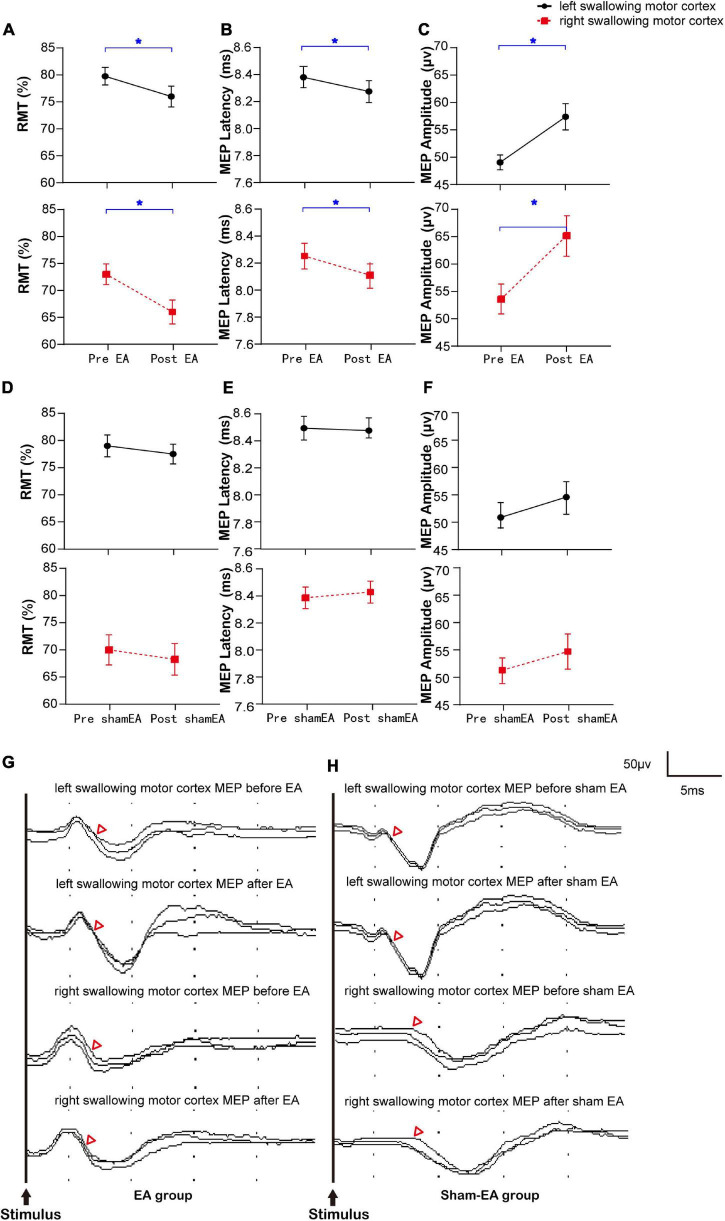
Bilateral RMT and MEP of the swallowing motor cortex before and after the intervention and representative MEP. **(A–C)** The RMT and MEP of the cerebral swallowing motor cortex before and after EA. **(D–F)** The RMT and MEP of the cerebral swallowing motor cortex before and after sham-EA. **(G,H)** Representative EMG from the bilateral swallowing motor cortices before and after the intervention. RMT, resting motor threshold; MEP, motor evoked potential; EA, electro-acupuncture; *P < 0.05; △ represents the latency of motor evoked potential.

The same sample was divided into an EA group and a sham-EA group to study the effects of EA on the brain. There were no significant between-group differences in sex, age, Kubota Water Swallow Test score, Mini-mental State Examination score, or body mass index (Details in Supplementary Appendix 4). The results of RMT, MEP latency, and MEP amplitude of the mylohyoid induced by bilateral stimulation of the swallowing motor cortex in the EA and sham-EA groups are shown in Figures 2A–F and Supplementary Appendix Table 3B, respectively. Figures 2G,H shows representative MEP from the bilateral swallowing motor cortices before and after the intervention. On comparing the changes in the RMT, MEP latency, and MEP amplitude of the contralateral mylohyoid innervated by the right and left swallowing motor cortex, the RMT was diminished after EA (R: 73.00 ± 1.90 to 66.00 ± 2.22, L: 79.75 ± 1.60 to 76.00 ± 1.94, Wilcoxon’s tests, *Z* = −2.839, *P* = 0.005; *Z* = −3.866, *P* = 0.0001, Figure 2A), the MEP latency was shortened after EA (R: 8.24 ± 0.10 to 8.10 ± 0.10, L: 8.36 ± 0.08 to 8.26 ± 0.08, Wilcoxon’s tests, *Z* = −3.041, *P* = 0.002; *Z* = −3.362, *P* = 0.001, Figure 2B), and the MEP amplitude was enlarged after EA (R: 54.06 ± 2.73 to 65.29 ± 3.72, L: 49.53 ± 1.36 to 57.84 ± 2.43, Wilcoxon’s tests, *Z* = −3.192, *P* = 0.002; *Z* = −2.763, *P* = 0.009, Figure 2C). However, these measures were not significantly different after sham-EA (Wilcoxon’s tests, *P* > 0.05, Figures 2D–F). Thus, the excitability of right and left swallowing motor cortices was only increased after EA intervention.

### Effect of Electro-Acupuncture on Lateralization of Swallowing Motor Cortex

**FIGURE 3:**
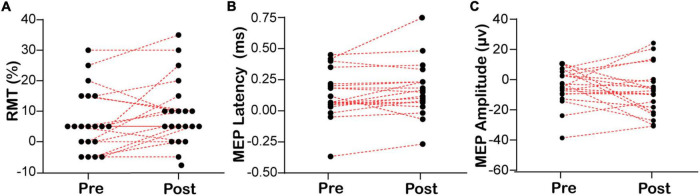
Lateralization of bilateral RMT and MEP before and after EA. **(A)** The lateralization of resting motor threshold. **(B,C)** The lateralization of motor evoked potential. RMT, resting motor threshold; MEP, motor evoked potential. RMT/MEP lateralization was calculated according to the following formula: RMT/MEP lateralization = left swallowing motor cortex RMT/MEP – right swallowing motor cortex RMT/MEP.

Based on the above findings that the excitability of the right swallowing motor cortex was higher than that of the left side in healthy subjects, we investigated if EA can regulate the lateralization of swallowing motor cortex excitability. The changes in the RMT, MEP latency, and MEP amplitude of the mylohyoid induced by the bilateral swallowing motor cortices in the EA and sham-EA groups are shown in Figure 3 and Supplementary Appendix Table 3C. There was no significant difference in the RMT (6.75 ± 2.27 to 10.00 ± 2.32, Wilcoxon’s tests, *Z* = −1.832, *P* = 0.067, Figure 3A) or MEP latency (0.12 ± 0.04 to 0.16 ± 0.05, Wilcoxon’s tests, *Z* = −1.419, *P* = 0.156, Figure 3B) of the contralateral mylohyoid innervated by the right swallowing motor cortex after EA, but there was a trend toward an increase in changes of RMT in the right swallowing motor cortex. In addition, since there is no significant change in the swallowing motor cortex excitability before and after the sham-EA, there was no significant difference in the lateralization of swallowing motor cortex excitability after the sham-EA (Supplementary Appendix Table 3D).

## Discussion

Our study revealed evidence of a dominant right-sided lateralization of swallowing motor cortex excitability in the healthy people at rest. We also found that EA at CV23 and GV16 can enhance excitability of the bilateral swallowing motor cortices, while the sham-EA could not. In addition, EA did not significantly change the lateralization of the swallowing motor cortex under physiological conditions.

Swallowing is a sensory-motor behavior regulated primarily by the brainstem and cerebral cortex (Ludlow, 2015). To date, the regulatory mechanism of human swallowing remains incompletely understood. With the development and wide application of many non-invasive human brain imaging techniques, positron emission tomography, functional MRI, magnetoencephalography, and TMS have been applied to study the cerebral motor cortex and swallowing (Hamdy et al., 1999; Suzuki et al., 2003). Some authors believe that the primary motor cortex is the initiating region for swallowing, whereas others believe that while the primary motor cortex is active during swallowing, it may play a more executive role, perhaps by balancing the excitatory and inhibitory mechanisms of the brainstem (Zald and Pardo, 1999; Mosier and Bereznaya, 2001; Furlong et al., 2004). Other studies have suggested that the motor cortex is involved in triggering the swallowing mechanism, and the motor cortex representation for swallowing displays territorial asymmetry (Mistry et al., 2007). These findings have led many researchers to infer a possible swallowing functional hemispheric dominance. Our study revealed that the excitability of the swallowing cortex is greater on the right side than on the left side by observing RMT and MEP, which indicates that the motor swallowing cortex is right-sided dominant.

Acupuncture originated in China 2,000 years ago as part of traditional Chinese medicine and is a minimally invasive therapy that regulates the human body (Kaptchuk, 2002). EA is an improved acupuncture therapy that stimulates acupoints by an electric current rather than manually. EA is widely used in clinical treatment and basic acupuncture research because of its controllable stimulation parameters and repeatability (Syuu et al., 2001; Elbasiouny et al., 2010). Acupuncture at CV23 and GV16 has been used to regulate swallowing function for thousands of years in China. CV23 is located between the thyroid cartilage and the hyoid bone, and deep tissue is innervated by branches of the hypoglossal and glossopharyngeal nerves (Shi et al., 2019). Human anatomy studies have confirmed that GV16 is located directly above the medulla oblongata and is innervated by the greater occipital nerve, accessory nerve, and cervical nerve (C1–C3). Some studies have shown that the afferent nerve of swallowing overlaps with the afferent nerve of CV23 and GV16 (Shi et al., 2019). It is believed that EA at CV23 and GV16 can stimulate the cervical nerve and hypoglossal nerve related to swallowing activities, enhance the excitability of swallowing afferent fibers, transmit impulses, and provide sensory feedback to the central nervous system (Ye et al., 2019). With the enhancement of sensory consciousness, the deglutition central pattern generator converts the excitatory information from the central and peripheral input into burst activity of motor neurons, which allows more new motion-projection areas to be created that evoke resting synapses to transmit nerve impulses (Guertin, 2013).

Our study confirmed that EA at CV23 and GV16 enhanced the excitability of the swallowing motor cortex at resting state by observing RMT and MEP, whereby there was an instantaneous increase of bilateral swallowing motor cortices excitability. Consistent with our study results, another study indicated that acupuncture therapy can modulate the corticomotoneuronal excitability and interhemispheric competition on healthy subjects, further enhance the excitability of the bilateral cortex (Yang et al., 2017). The symptoms of patients with dysphagia may be improved by increasing the excitability of the swallowing cortex, in other words, the changes in excitability would drive swallowing recovery (Hamdy et al., 1997; Sawan et al., 2020). Clinical studies have shown that acupuncture can effectively improve dysphagia (Lu et al., 2021), which may be related to EA at CV23 and GV16 enhanced the excitability of the swallowing motor cortex at resting state.

Concerning the lateralization of the swallowing motor cortex, we found that the lateralization of the latent period of RMT and MEPs of the swallowing motor cortex after the intervention of EA tended to further strengthen on the dominant side, but did not really change the inherent laterality of the swallowing motor cortex. McCambridge et al. (2019) demonstrated that EA has no significant regulatory effect on the cerebral cortex in healthy adults. Using TMS-EMG, Yang et al. (2017) found that acupuncture can increase the excitability of the primary motor cortex of the affected side of the brain in patients with stroke, while reducing contralateral primary motor cortex excitability. Our future work will focus on whether lateralization could influence the speed and degree of recovery in patients with post-stroke dysphagia, and the regulatory effect of EA on the bilateral swallowing cortex of these patients.

## Limitations

Our study has some limitations that should be considered. First, the study subjects were healthy right-handed people, although some studies have proposed that handedness has no effect on lateralization of the cerebral cortex, this still needs to be formally proved by including more left-handed subjects in future studies. Second, the limited sample size could have restricted the detection of statistically significant effects. Third, the duration of excitability changes by EA were not examined. Fourth, due to the limitations of this technology, although we tried to ensure that the stimulation site was in the swallowing motor cortex of the precentral gyrus, strictly speaking, such positioning and stimulation methods were still relatively rough. Fifth, the physiological state of the cerebral cortex may not be consistent with pathological conditions. Thus, further studies are needed to demonstrate the relationship between excitability of the bilateral swallowing cortex and swallowing function.

## Conclusion

The right swallowing motor cortex of healthy subjects is more excitable than that of the left at resting state. Thus, we found that lateralization is present in the swallowing motor cortex of healthy people, which indicates a hemispheric dominance of swallowing predominates in the right swallowing motor cortex. In addition, EA at CV23 and GV16 was found to instantly promote excitability of the bilateral swallowing motor cortices. Moreover, although there was no significant difference in lateralization, we found an increasing trend that EA could regulate the lateralization of human swallowing motor cortex excitability. Our future work will investigate the effect of EA on lateralization of the swallowing motor cortex excitability in patients with dysphagia.

## Data Availability Statement

The raw data supporting the conclusions of this article will be made available by the correspondence authors, without undue reservation.

## Ethics Statement

The studies involving human participants were reviewed and approved by China Ethics Committee for Registering Clinical Trials (reference number ChiECRCT-20170038). The patients/participants provided their written informed consent to participate in this study.

## Author Contributions

XT: conceptualization, methodology, software, formal analysis, investigation, writing – original draft, writing review and editing, and visualization. MX: methodology, formal analysis, writing original draft, and visualization. JZ and JW: investigation, writing – original draft, and writing – review and editing. JS: writing original draft, writing – review and editing, and visualization. YZ: software, investigation, writing – original draft, and writing – review and editing. JX, LY, and HL: investigation and writing – review and editing. YY: methodology, investigation, and writing – review and editing. JO and SY: writing – review and editing. YL: writing – original draft and writing – review and editing. LL: conceptualization and writing – review and editing. NX: conceptualization, writing – review and editing, and funding acquisition. LW: conceptualization, resources, writing – review and editing, supervision, and funding acquisition. All authors contributed to the article and approved the submitted version.

## Conflict of Interest

The authors declare that the research was conducted in the absence of any commercial or financial relationships that could be construed as a potential conflict of interest.

## Publisher’s Note

All claims expressed in this article are solely those of the authors and do not necessarily represent those of their affiliated organizations, or those of the publisher, the editors and the reviewers. Any product that may be evaluated in this article, or claim that may be made by its manufacturer, is not guaranteed or endorsed by the publisher.

## Abbreviations

EA: electro-acupuncture
EMG: electromyography
MEP: motor evoked potential
MRI: magnetic resonance image
RMT: resting motor threshold
TMS: transcranial magnetic stimulation.
