# Supplementary material for: Effect of Electro-Acupuncture on Lateralization of the Human Swallowing Motor Cortex Excitability by Navigation-Transcranial Magnetic Stimulation-Electromyography
Source: Front Behav Neurosci. 2022 Feb 24;16:808789. doi: 10.3389/fnbeh.2022.808789 (PMC8911038; doi:10.3389/fnbeh.2022.808789)
Supplement: Supplementary file 1 [file Presentation_1.zip › Appendix 3- Data table.pdf]

Appendix 3

Appendix 3.Table A The RMT and MEP of the contralateral mylohyoid innervated by the bilateral swallowing motor cortex ($\bar{x}\pm$ SEM, n = 40)

	Left swallowing cortex	Right swallowing cortex	*P* Value[a]
RMT(%)	79.38 ± 1.27	71.50 ± 1.67**	< 0.001
MEP latency (ms)	8.40 ± 0.06	8.30 ± 0.06*	0.041
MEP amplitude (μv)	50.15 ± 1.23	52.68 ± 1.76	0.422

RMT: resting motor threshold; MEP: motor evoked potential.

[a] *P* values were based on analysis of Mann-Whitney U test analysis. * *P* values<0.05, ** *P* values<0.01.

Appendix 3.Table B Bilateral RMT and MEP of the swallowing motor cortex before and after the intervention and representative MEP（$\bar{X}\pm$ SEM, n=20 in each group）

	EA			Sham-EA		
	Pro	Post	*P* Value[b]	Pro	Post	*P* Value[b]
RMT-L(%)	79.75 ± 1.60	76.00 ± 1.94	0.005	79.00 ± 2.01	77.50 ± 1.83	0.083
RMT-R(%)	73.00 ± 1.90	66.00 ± 2.22	< 0.001	70.00 ± 2.76	68.25 ± 2.91	0.112
MEP latency -L(ms)	8.36 ± 0.08	8.26 ± 0.08	0.002	8.47 ± 0.09	8.45 ± 0.09	0.717
MEP latency -R(ms)	8.24 ± 0.10	8.10 ± 0.10	0.001	8.36 ± 0.08	8.40 ± 0.08	0.266
MEP amplitude -L (μv)	49.53 ± 1.36	57.84 ± 2.43	0.002	50.78 ± 2.07	54.55 ± 2.82	0.191
MEP amplitude -R (μv)	54.06 ± 2.73	65.29 ± 3.72	0.009	51.30 ± 2.25	54.69 ± 3.23	0.279

L: left swallowing motor cortex; R: right swallowing motor cortex; RMT: resting motor threshold; MEP: motor evoked potential. [b] *P* values were based on analysis of Wilcoxon test analysis which compared the differences in bilateral swallowing cortex excitability before and after the intervention（EA or sham-EA).

Appendix 3.Table C Lateralization of bilateral RMT and MEP before and after EA

（$\overline{X}$± SEM, n=20）

	Pro[c]	Post[c]	*P* Value[b]
RMT(%)	6.75 ± 2.27	10.00 ± 2.32	0.067
MEP latency (ms)	0.12 ± 0.04	0.16 ± 0.05	0.156
MEP amplitude (μv)	-4.53 ± 2.69	-7.45 ± 3.56	0.526

RMT: resting motor threshold; MEP: motor evoked potential. [b] *P* values were based on analysis of Wilcoxon test analysis which compared the differences in bilateral swallowing cortex excitability before and after the electro-acupuncture. RMT/MEP lateralization was calculated according to the following formula: RMT/MEP lateralization = left swallowing motor cortex RMT/MEP - right swallowing motor cortex RMT/MEP.

Appendix 3.Table D Lateralization of bilateral RMT and MEP before and after sham-EA

（$\overline{X}$± SEM, n=20）

	Pro[c]	Post[c]	*P* Value[b]
RMT(%)	9.00 ± 2.48	9.25 ± 2.67	0.822
MEP latency (ms)	0.10 ± 0.04	-0.01 ± 0.09	0.911
MEP amplitude (μv)	-0.53 ± 2.34	-0.14 ± 3.69	0.211

RMT: resting motor threshold; MEP: motor evoked potential. [b] *P* values were based on analysis of Wilcoxon test analysis which compared the differences in bilateral swallowing cortex excitability before and after the electro-acupuncture. RMT/MEP lateralization was calculated according to the following formula: RMT/MEP lateralization = left swallowing motor cortex RMT/MEP - right swallowing motor cortex RMT/MEP.
