# Supplementary material for: Effect of Electro-Acupuncture on Lateralization of the Human Swallowing Motor Cortex Excitability by Navigation-Transcranial Magnetic Stimulation-Electromyography
Source: Front Behav Neurosci. 2022 Feb 24;16:808789. doi: 10.3389/fnbeh.2022.808789 (PMC8911038; doi:10.3389/fnbeh.2022.808789)
Supplement: Supplementary file 1 [file Presentation_1.zip › Appendix 4- Demographic variable table for each group.pdf]

Appendix 4 Demographic variable table for each group（$\overline{X}$ ±SD）

Variables	EA group（n=20）	Sham-EA group（n=20）	*P* Value
Age	21.45±1.96	21.85±1.60	0.484
Sex(Female/Male)	10/10	10/10	/
BMI（kg/㎡）	19.92±1.87	20.89±2.31	0.154
Kubota Water Swallow Test score	1.00±0.00	1.00±0.00	/
MMSE score	30.00±0.00	30.00±0.00	/

BMI:body mass index, MMSE:Mini-mental State Examination, *P* values were based on analysis of unpaired t test analysis.
