# Supplementary figures and images for: Effect of Electro-Acupuncture on Lateralization of the Human Swallowing Motor Cortex Excitability by Navigation-Transcranial Magnetic Stimulation-Electromyography
Source: Front Behav Neurosci. 2022 Feb 24;16:808789. doi: 10.3389/fnbeh.2022.808789 (PMC8911038; doi:10.3389/fnbeh.2022.808789)

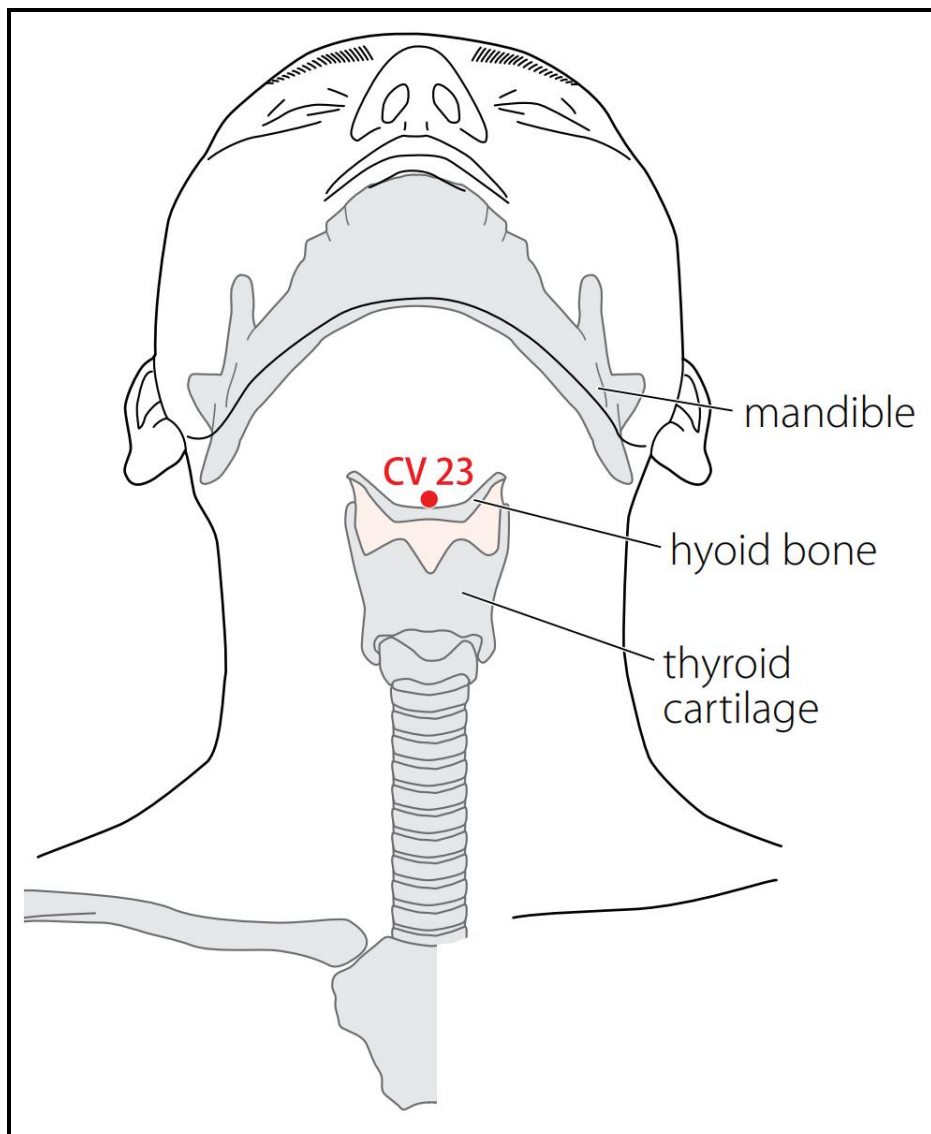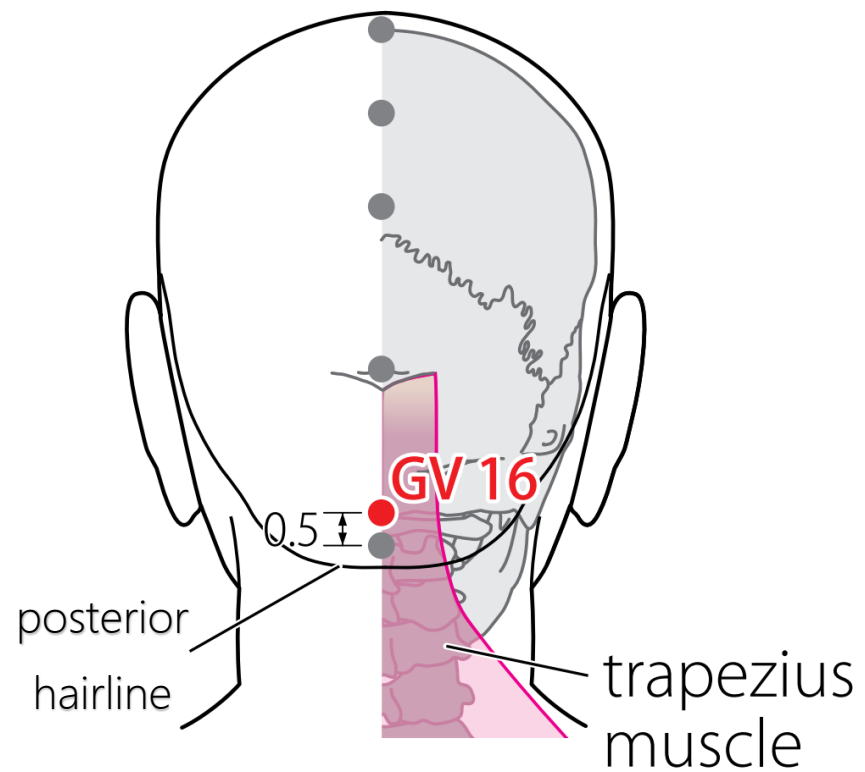

Supplement: Supplementary file 1 [file Presentation_1.zip › Appendix 1- Location of the CV23 and GV16.pdf]

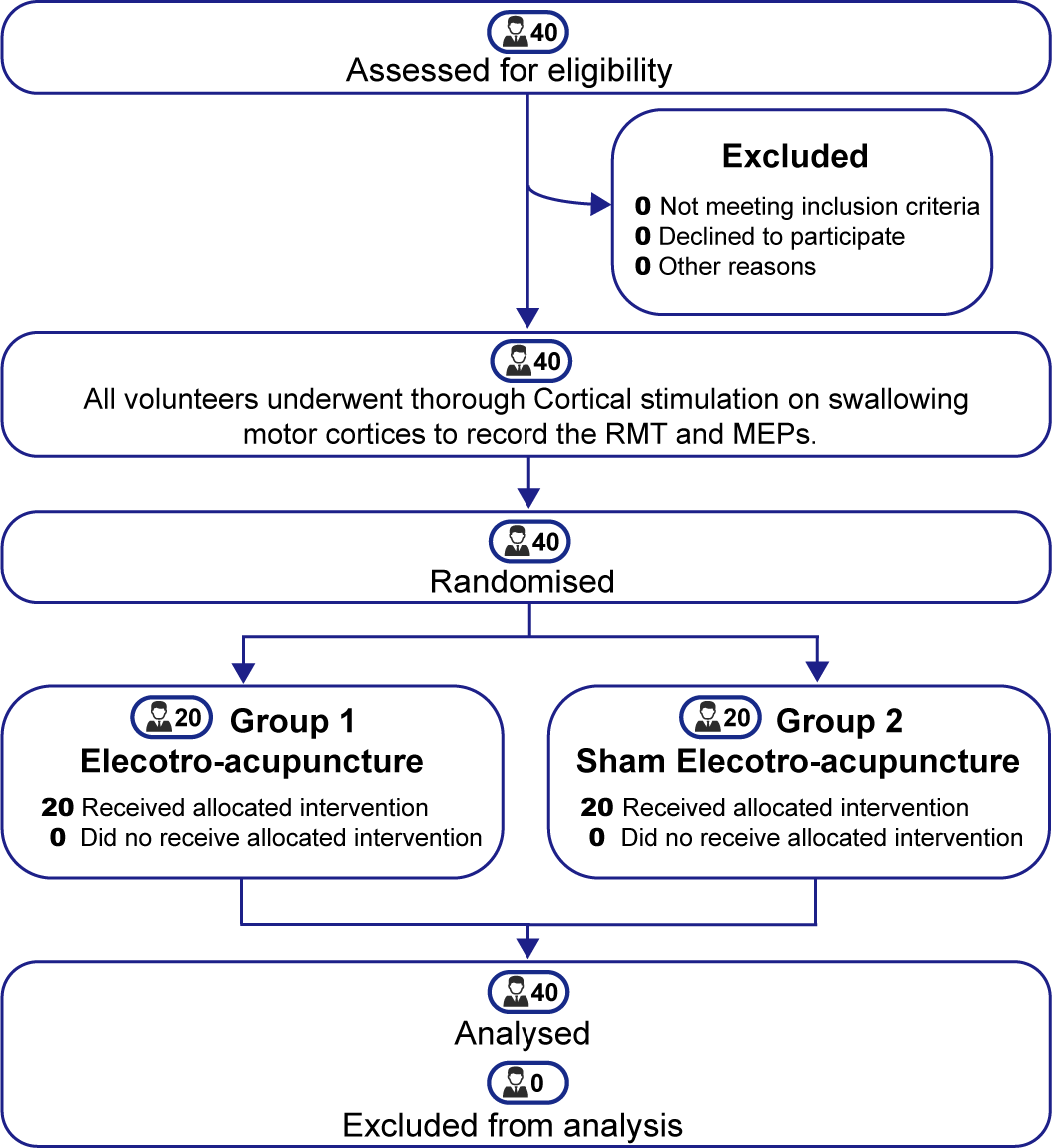

Supplement: Supplementary file 1 [file Presentation_1.zip › Appendix 2- Participant flow diagram.tif]
